# Association between cannabis use and symptoms of psychosis: a mega-analysis

**DOI:** 10.1192/j.eurpsy.2023.1308

**Published:** 2023-07-19

**Authors:** M. Argote, B. Rolland, G. Sescousse, J. Brunelin, E. Fakra, M. Nourredine, R. Jardri

**Affiliations:** 1 PSYR2 team, CH Le Vinatier, Lyon; 2 Pôle universitaire de psychiatrie, CHU Saint-Etienne, Saint-Etienne; 3 Service de biostatistiques, Hospices civiles de Lyon, Lyon; 4 Centre Hospitalier de Lille, Lille, France

## Abstract

**Introduction:**

It remains debated in the scientific literature whether cannabis aggravates psychotic symptoms or is used as a self-medication. Regular cannabis use (RCU) was found associated with the severity of positive symptoms of psychosis i.e., delusion or hallucinations. However, the association with negative symptoms, i.e. blunted affect or social withdrawal, is less straightforward. Confounding variables such as the criteria for other Substance Use Disorders (SUDs), illness duration, or socio-demographic features can interfere with the appraisal of effect of RCU on these symptoms. Studies are rarely adjusted for these variables and no adjustment can be performed in meta-analyses which use aggregated data. For these reasons, we decided to conduct a mega-analysis based on individual patient data (IPD) allowing to control for theses variables and isolate the specific association between RCU and the severity of symptoms.

**Objectives:**

Investigate the association between RCU on the positive, negative, general, and disorganized symptoms of psychosis as assessed by the Positive and Negative Syndrome Scale (PANSS), accounting for individual-level confounding variables.

**Methods:**

IPD were requested by email to corresponding authors of published articles that measured RCU and PANSS scores in subjects with schizophrenia-spectrum disorders, based on a screening process on PubMed, ScienceDirect and PsycINFO databases. A two-stage random effect multivariate IPD meta-analysis was then performed, to isolate the direct association between RCU and the ‘positive’, ‘negative’, and ‘general’ dimensions of schizophrenia-spectrum disorders. Confounding variables were included in the models when available in the original dataset.

**Results:**

65 publications were eligible for inclusion. 18 authors agreed to provide their IPD. A total of 16 datasets were usable, regrouping 3,346 individual participant data, with 2,827 complete cases. Regression coefficients extracted after the first stage were adjusted for at least sex and age across all studies. RCU was found to be significantly associated with heightened positive symptoms severity (MD = 0.41, 95% CI [0.0; 0.82], p = 0.04), whereas it appeared significantly associated with less severe negative symptoms (MD = -0.63, 95% CI [-1.1; -0.17], p = 0.008). No significant association was found between RCU and general symptoms (MD = -0.24, 95% CI [-069; 0.21]; p = 0.29), as well as disorganization (MD = -0.08, 05%CI [-0.47; 0.35], p = 0.63).

**Conclusions:**

Our results allow for a general and subtle overview of the association of RCU with symptoms of psychosis. Our findings suggest a double and paradoxical effect of cannabis, which could both exacerbate positive symptoms and alleviate negative symptoms. This supports both the hypotheses of a disease aggravator and self-medication.

*Results change as we receive datasets from collaborating authors and could continue to change as not all authors sent their datasets yet.*

**Disclosure of Interest:**

None Declared

